# Biopsychosocial exposure to the COVID-19 pandemic and the relative risk of schizophrenia: Interrupted time-series analysis of a nationally representative sample

**DOI:** 10.1192/j.eurpsy.2021.2245

**Published:** 2022-01-24

**Authors:** Yael Travis-Lumer, Arad Kodesh, Yair Goldberg, Abraham Reichenberg, Sophia Frangou, Stephen Z. Levine

**Affiliations:** 1 The Faculty of Industrial Engineering and Management, Technion - Israel Institute of Technology, Haifa, Israel; 2 Department of Community Mental Health, University of Haifa, Haifa, Israel; 3 Mental Health Department Meuhedet Health Services, Tel Aviv, Israel; 4 Department of Psychiatry, Icahn School of Medicine at Mount Sinai, New York, New York, USA; 5 Djavad Mowafaghian Centre for Brain Health, Department of Psychiatry, University of British Columbia, Vancouver, British Columbia, Canada

**Keywords:** COVID-19, epidemiology, public health, schizophrenia

## Abstract

**Background:**

Studies of COVID-19 pandemic biopsychosocial exposure and schizophrenia risk showed contradictory results, were undertaken early in the pandemic, and did not consider lockdowns or COVID-19 infection. Hence, we examined the association between COVID-19 biopsychosocial exposure and incident schizophrenia.

**Methods:**

An interrupted time-series study design was implemented based on Israeli electronic health records from 2013 to 2021 with national coverage. The period coinciding with the COVID-19 pandemic biopsychosocial exposures from March 2020 to February 2021 was classified as exposed, otherwise unexposed. The effect of the COVID-19 pandemic on incident schizophrenia was quantified by fitting a Poisson regression and modeling the relative risk (RR) and corresponding 95% confidence intervals (CI). Three scenarios were projected from the third lockdown to 10 months to forecast incident schizophrenia rates and their associated 95% prediction intervals (PI).

**Results:**

The total population (N = 736,356) yielded 4,310 cases of incident schizophrenia over time. The primary analysis showed that the period exposed to the COVID-19 pandemic was associated with a reduced RR (RR = 0.81, 95% CI = 0.73, 0.91, p < 0.001). This conclusion was supported in 12 sensitivity analyses, including scrutinizing lockdowns and COVID-19 infection status. Two of three forecast scenarios projected an incident increase (6.74, 95% PI = 5.80, 7.84; 7.40, 95% PI = 6.36, 8.60).

**Conclusions:**

The reduced risk of schizophrenia during the pandemic suggests no immediate triggering of new onsets either by the virus or the pandemic-induced psychosocial adversities. Once restrictions are lifted, the increased projected presentations have implications for clinicians and healthcare policy.

## Introduction

The Coronavirus disease 2019 (COVID-19) is the most recent of three deadly coronaviruses to emerge in humans in the past decade [1]. Globally, by January, 25, 2022, the World Health Organization received reports of 349,641,119 confirmed cases of COVID-19, including 5,592,266 deaths since the onset of the outbreak in December 2019 [2,3]. Governments worldwide implemented public health COVID-19 attenuation policies that consist of social restrictions (including lockdown periods), which have proved efficacious in indenting COVID-19 related infection and death [4–7].

However, these strategies and the disease itself have acted as a catalyst for multiple biopsychosocial adversities. We use the qualifier “biopsychosocial capture” to capture the experiences of the COVID-19 pandemic. These experiences include the biological exposure to the COVID-19 infection and the social and psychological experiences of the COVID-19 attenuation policies, which include lockdown, social distancing, loneliness, financial loss, unemployment [8], secondary health effects (e.g., weight gain) [9], and crime [10]. Many of these biopsychosocial pandemic adversities may be risk factors for psychosis, for example, loneliness [11] and social defeat [12]. Although research demonstrates that schizophrenia is a risk factor for COVID-19 infection [13,14], the reverse is etiologically plausible. Clinical observations [15–18], theories [19,20], and research [21–23] imply that the multiple biopsychosocial COVID-19 pandemic adversities may increase the risk of schizophrenia. However, the available data are contradictory, indicating both COVID-19 related reductions [24,25] and increases [26] in the rate of incident schizophrenia compared to the pre-exposure periods.

To date, four studies have examined the effect of the COVID-19 pandemic on schizophrenia. In the Rhineland region of Germany, based on inpatient service utilization for a range of psychiatric disorders during the first 3 months of the pandemic, results suggested reduced service utilization across all disorders, which was less pronounced for schizophrenia but increased in presentations with atypical psychotic features [25]. Similarly, based on electronic medical records during the first home-confinement period in the UK, a significant reduction in all mental health-related primary and secondary care contacts, including psychosis, was observed [24]. In a survey of three European countries during confinement, daily fluctuations of brief psychotic-like experiences increased with associated country-specific COVID-19 deaths [23]. In China, outpatient registry data suggested that the period of COVID-19 was associated with an increased risk of schizophrenia compared to beforehand [26]. These early studies were undertaken early in the pandemic and so could not capture the protracted nature of the multiple biopsychosocial COVID-19 exposures (e.g., cumulative multiple stressors and infection). The studies lack information about incident cases and so we are unable to address whether the biopsychosocial exposures of the COVID-19 pandemic could trigger the onset of schizophrenia. Importantly, their approach did not enable inferences about evolving mental health needs that are critical for planning service provision.

Two-hit models may offer an avenue to explain the onset of the clinical expression of schizophrenia. In these models, first, genetic or environmental insults disrupt early central nervous system development. These early insults invoke long-term vulnerability to a “second hit” that triggers the onset of schizophrenia [27,28]. Plausibly, the biopsychosocial exposures of the COVID-19 pandemic triggered a “second hit.”

In response, the present study used a nationally representative sample to test the “trigger” hypothesis of COVID-19 biopsychosocial stress for schizophrenia. Statistical modeling of the effect of the pandemic was supplemented by robust sensitivity testing to examine the contributions of sociodemographic factors, methodological artifacts, lockdowns, and COVID-19 infection. Importantly, we present data on the predicted post-pandemic incidence of schizophrenia under three different forecast scenarios to assist in planning for emerging health care needs.

## Methods

### Population

The study source population was nationwide coverage of all Meuhedet members aged between 15 and 64 years. Specifically, electronic health registry data (which are continuously collected and updated) were examed on 736,356 individuals (male *N* = 371,365, 50.4%; female *N* = 364,991, 49.6%) aged between 15 and 64 years at the Meuhedet. Meuhedet Healthcare Services (hereafter Meuhedet) is one of the four Israeli health maintenance organizations (HMOs), covered by the Israeli HMO legislation and serves 14% of the total population of Israel nationwide.

The National Health Insurance Law in Israel dictates that four nonprofit public HMOs provide healthcare services to the entire population [29]. This legislation specifies that all HMOs must offer nationwide services that do not differ financially or in service provision. By law, each Israeli citizen must choose to join a single HMO. HMOs cannot deny residents membership based on location, demographic or medical characteristics (i.e., age, minority-group status, and medical history). Accordingly, noninclusion by an HMO (and hence, sample selection) would violate Israeli legislation. The study received approval from the Meuhedet associated Helsinki Institutional Review Board with a waiver of informed consent.

### Exposure

The first confirmed case of COVID-19 in Israel was on February 27, 2020, and the first lockdown started on March 14, 2020. Accordingly, the interval from January 1, 2013 to February 1, 2020 was classified as the “unexposed period,” and the interval between March 1, 2020 and ending on February 1, 2021 was designated as the “exposed period.” The exposed period covered three COVID-19 waves of government-imposed national COVID-19 attenuation strategies (e.g., lockdowns). These COVID-19 pandemic policy restrictions imposed in Israel during the study period are documented in Table 1 and are based on the Oxford COVID-19 Government Response Tracker [30]. These include the closure of all schools and workplaces, restricted gatherings, staying at home, internal movement restrictions, and banning international travel.

**Table 1.** COVID-19 Israel policy restrictions.

Interval	Date begins	Date ends	Restrictions
Pre-lockdown 1	2020-03-01	2020-03-13	International travel banned
Lockdown 1	2020-03-14	2020-04-30	All schools closed, workplaces closed, restrictions on gatherings, stay at home, internal movement restrictions, and international travel banned
Post-lockdown 1	2020-05-01	2020-09-18	Restrictions on gatherings, stay at home, internal movement restrictions, and international travel banned
Lockdown 2	2020-09-18	2020-10-17	All schools closed, workplaces closed, restrictions on gatherings, stay at home, and internal movement restrictions
Post-lockdown 2	2020-10-18	2020-12-26	All schools closed, restrictions on gatherings, stay at home, internal movement restrictions, and international travel banned
Lockdown 3	2020-12-27	2021-02-07	All schools closed, workplaces closed, restrictions on gatherings, stay at home, internal movement restrictions, and international travel banned
Entire COVID-19 period	2020-03-01	2021-02-28	Public events canceled

*Note:* All schools closed except nurseries. Workplaces closed for all but essential workplaces. Based on the Oxford COVID-19 Government Response Tracker [30] for each interval and each restriction, we compared the mode value of the restriction to a threshold to classify whether the restriction occurred during the interval. For most restrictions, we used the Oxford COVID-19 Government Response Tracker threshold value of two, whereas for international travel ban, restrictions on gatherings and workplaces closed, we used a threshold value of three.

### Ascertainment of schizophrenia

Monthly incident rates of schizophrenia (including spectrum disorders) were ascertained from ICD codes (version 9: 295–299; version 10: F20–F29) based on diagnoses confirmed by a board confirmed specialist. These diagnoses in this data source were used in prior schizophrenia research [31], and the healthcare setting in Israel prevents noninsurance of persons with schizophrenia.

### Statistical analysis

We used interrupted time series (ITS, Supplementary Figure S1) [32–34], a quasi-experimental study design [35], to compare the monthly incident schizophrenia rates between the exposed and unexposed periods [24–26]. Schizophrenia spectrum disorders trends were examined for distinct changes from preexisting trends, termed a counterfactual. This study design is instrumental when retrospective evaluations of population-level interventions are required. The ITS modeled time (as a monthly sequence during the entire 7-year period of the study period), exposure period (i.e., unexposed or exposed), and their interaction. Additional covariates were an offset term to model event rates and seasonal Fourier terms to model the seasonal factors.

For the primary analysis, to compare the intervals unexposed and exposed to the COVID-19 pandemic, we fitted a Poisson regression model and quantified the relative risk (RR) and associated 95% confidence intervals (CI) of the total monthly incident schizophrenia rate. The RR compares the model predictions for the exposed period to the model predictions for the same period but under the assumption that COVID-19 had not occurred (i.e., the counterfactual), accounting for the study covariates.

For public health policy post-wave three, we extended the primary Poisson regression model to forecast post-wave three schizophrenia incident rates from March 1, 2021 to December 1, 2021, under three scenarios (a) assuming no ongoing effects of the COVID-19 pandemic; (b) assuming ongoing effects of the COVID-19 pandemic; and (c) were estimated based on the periods both before and during the COVID-19 pandemic.

The robustness of the primary analysis results was challenged in 12 sensitivity analyses. The first set focused on sex and socioeconomic status (SES; defined in Supplementary Material) which are known modifiers of schizophrenia risk [36,37]. The second set examined potential methodological artifacts, including seasonal decompositions, and unit of time from a month to a 15-day interval to test for the potential artifact of aggregation. We used the incident rates of schizophrenia before and during the Gaza war as a negative control group to consider potential confounding and bias [38]. This war (July 8–August 24, 2014) may also be considered as a “traumatic” event by the Israeli population due to military and civilian fatalities and severe rocket bombing. The final series of sensitivity analyses focused on the impact of the severity of social restriction and of COVID-19 infection status. Accordingly, we tested for potential differences in schizophrenia incident rates during the lockdown-on and lockdown-off periods based on 15-day intervals and between COVID-19 positive and negative cases for equality of proportions [39]. Finally, we conducted an additional ITS, like the primary analysis, restricted only to individuals who did not test positive for COVID-19 in each month. Analyses were implemented in R [40], with the packages forecast [41], and ggplot2 [42].

## Results

**Figure 1.**
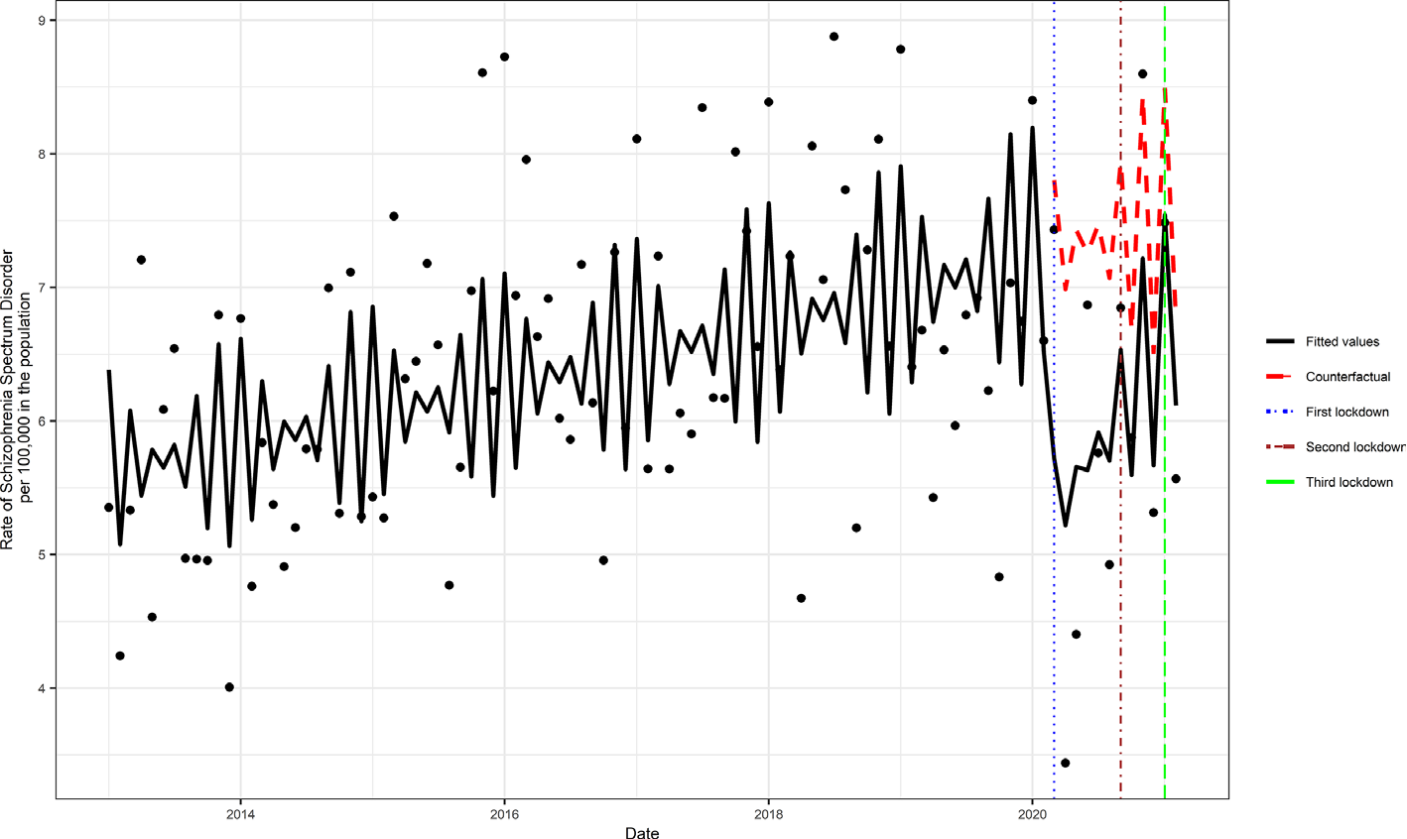
Comparison of the periods with and without Covid-19 pandemic exposure. The counterfactual refers to the predicted values had no COVID-19 occurred, and the fitted values are estimated based on the Poisson regression model.

We identified a total of 4,310 incident cases of schizophrenia (total cumulative rate per 100,000 = 585.31, 95% CI = 567.97, 603.06) across the study period. The monthly incident rate of schizophrenia across the study intervals ranged from 3.40 (CI 2.20, 5.01) to 8.56 (CI 6.57, 10.95) per 100,000 in the population. Although the trend in the incident rate in schizophrenia increased before the COVID-19 pandemic, the primary analysis showed that during the exposed period there was a statistically significant (RR = 0.81, 95% CI = 0.73, 0.91, *p* < 0.001) reduction in the incidence of schizophrenia (Figure 1). The model assumptions were not violated by residual autocorrelation and residual partial autocorrelations (Supplementary Figure S2).

**Figure 2.**
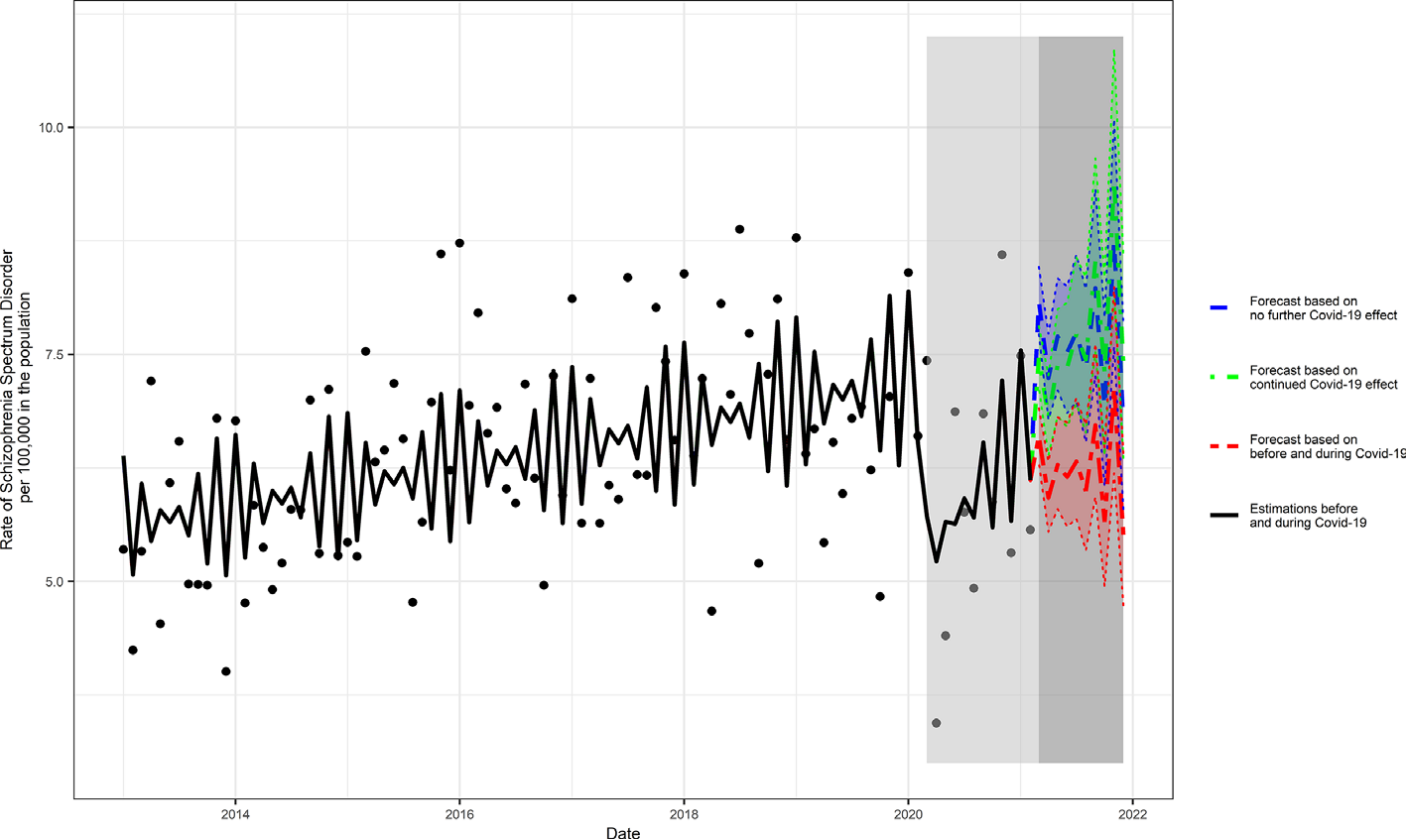
Three scenarios of forecasted COVID-19 pandemic effects on the rate of schizophrenia. The following three scenarios were scrutinized (a) assuming no ongoing effects of the COVID-19 pandemic; (b) assuming ongoing effects of the COVID-19 pandemic; and (c) based on the intervals before and during COVID-19 pandemic.

The median incidence rate of schizophrenia during the unexposed period was 6.43 per 100,000 in the population. Predicted 10-month estimates of schizophrenia incidence post-March 2021 ranged from 6.74 (95% prediction intervals [PI] = 5.80, 7.84), assuming no ongoing COVID-19 pandemic effect, to 7.40 (95% PI = 6.36, 8.60) assuming an ongoing pandemic effect; the scenario based on the intervals before and during the COVID-19 pandemic exposure yielded an estimate of 5.51 (95% PI = 4.74, 6.42) (Figure 2 and Supplementary Table S1).

### Sensitivity analysis

Sensitivity analyses restricted to males, females as well as low, medium, and high SES confirmed the results of the primary analyses (Supplementary Table S2 and Supplementary Figures S3 and S4). Seasonal adjustments (Supplementary Figure S5) and changing the underlying time scale to 15-day intervals (Supplementary Figure S6, top panel) yielded comparable point precision estimates to the primary analysis. The Gaza war had no statistically significant effect on schizophrenia incidence rates, whereas the COVID-19 pandemic had a statistically significant decrease in schizophrenia incidence rates (Supplementary Figure S7 and Supplementary Table S2).

No differences in the incidence of schizophrenia were noted when comparing the lockdown-on and lockdown-off periods (*p* > 0.05) (Supplementary Figure S8 and Supplementary Table S2). Similarly, no difference in the proportions of the incidence of schizophrenia was noted when COVID-19 positive cases were compared to those who were COVID-19 negative (difference = 10^−5^*[−5.13], 95% CI = 10*^−5^ [−7.6,−2.66], *p*-value = 0.06). Third, sensitivity analysis restricted only to those with no evidence of COVID-19 infection yielded comparable point-precision estimates to the primary analysis (Supplementary Figure S9 and Supplementary Table S2).

## Discussion

We leveraged electronic medical record data collected using an epidemiological framework with national coverage to conduct an ITS analysis to test for the effect of biopsychosocial exposure to the COVID-19 pandemic on the incident rate of schizophrenia. To our knowledge, this is the first study to examine incident rates throughout the three waves of the pandemic while addressing sociodemographic features, the severity of social restrictions, and the COVID-19 infection status.

Despite the increasing time trend during the unexposed period before the pandemic, we found that the schizophrenia incidence rate dropped during the COVID-19 pandemic. Forecasting scenarios indicated that after COVID-19 pandemic-related mitigation strategies are lifted, the incidence of schizophrenia is likely to increase over 10 months.

The forecasting models suggest that the most likely future outcome is that the incidence of schizophrenia will increase even if there are no lingering biopsychosocial pandemic effects. This may be relevant, as most social mitigation strategies were lifted within Israel at the end of the data collection for this study. More than half of the Israeli population is now fully vaccinated, and economic recovery is expected, at least in the immediate aftermath of the third wave of the pandemic [43]. Hence, the end of the pandemic restrictions may signify a period of increased service needs for psychosis. This result may or may not generalize to other nations that are without restrictions at this time (e.g., the UK).

A unique feature of our study was the scrutiny of different COVID-19 pandemic related biopsychosocial exposures on the schizophrenia rate in sensitivity analyses. The results of sensitivity analysis included analyses restricted to different SES groups that replicated the primary results, analysis restricted to non-COVID-19 cases which replicated the primary results, COVID-19 lockdown status that given the exposed period had a null impact on the schizophrenia rate, and a negative control period of war that had a null impact on the schizophrenia rate. The current findings of different effects of the Gaza war and the COVID-19 pandemic periods suggest that it is inappropriate to generalize between large-scale traumatic events. Nonetheless, future research is warranted to compare the COVID-19 pandemic with other disasters. Collectively, it appears that the unique psychosocial factors, rather than the infection or lockdown of the COVID-19 pandemic, mainly impacted the schizophrenia rate.

The reduction in the incidence of schizophrenia during the pandemic may reflect reduced service use because of social restrictions and hence reduced opportunities for diagnostic assessments. This interpretation aligns with evidence from other studies that have noted an overall reduction of health service contacts across both physical and mental diagnoses [24]. Also, during the pandemic, the nature of health consultations changed with a rapid transition to digital platform use. It is theoretically possible that diagnoses of psychosis may have been missed because of this change of format. Other possibilities involve changes in family function during the pandemic, with families spending more time together than they would typically before the social restriction policies were in place. Furthermore, new-onset primarily affects young people, many of whom may have had to remain or return to their nuclear families as universities closed to in-person teaching or job opportunities away from home became scarce. At least for some young people, these changes may have been protective. However, causal mechanistic insights are not possible based on observational data alone but suggest important avenues for future research. Further research from other countries is required to test the generalizability of the present findings, although the initial available suggests similar trends, at least regarding mental health service contacts.

### Limitations

Causal conclusions are not possible based on observational data like ours, and it is not possible to eliminate residual confounders (e.g., occupations at risk). However, we examined groups with possibly differential schizophrenia risks, used a quasi-experimental study design, and an experiment of schizophrenia risk with COVID-19 would be unethical. Second, circumstances specific to Israel limit generalizing our results to other nations. For instance, in the first wave, Israel had a comparatively low COVID-19 mortality rate, a high number of COVID-19 cases, and entered the second wave and lockdown sooner than other nations [44]. Nonetheless, this limitation is balanced by the results considering three waves of COVID-19, and COVID-19 infection status.

Our results, for the first time, argue against COVID-19 infection having a direct pathogenetic effect for psychosis. It is plausible that a delayed effect occurred in the development of schizophrenia. We could not address this possibility owing to a lack of follow-up. Thus the results presented here are not conclusive, and replication is warranted. Similarly, owing to policy changes to address and variants of COVID-19, continued monitoring of the incidence of schizophrenia and related disorders is required to test for the possibility of a delayed direct effect and to test the accuracy of our predictions.

We implemented a quasi-experimental study design of unselected nationally representative cohort with little missing data, where the biases of selection and attrition are unlikely to explain our findings. The primary analysis reinforced by rigorous sensitivity analyses showed that exposure to the psychosocial adversities of the COVID-19 pandemic was associated with a reduced RR of schizophrenia. However, within 10 months after the COVID-19 pandemic and without COVID-19 restrictions in place, it is forecast that the schizophrenia rate will increase.

## Data Availability

Data access is possible following the processing of a reasonable request.

## Supplementary Materials

To view supplementary material for this article, please visit http://doi.org/10.1192/j.eurpsy.2021.2245. click here to view supplementary material

## References

[1] Fung TS, Liu DX. Similarities and dissimilarities of COVID-19 and other coronavirus diseases. Annu Rev Microbiol. 2021;75:19–47. doi:10.1146/annurev-micro-110520-023212. 33492978

[2] World Health Organization. WHO coronavirus disease (COVID-19) dashboard; 2020.

[3] Dong E, Du H, Gardner L. An interactive web-based dashboard to track COVID-19 in real time. Lancet Infect Dis. 2020;20:533–4. doi:10.1016/S1473-3099(20)30120-1. 32087114 PMC7159018

[4] Haug N, Geyrhofer L, Londei A, Dervic E, Desvars-Larrive A, Loreto V, et al. Ranking the effectiveness of worldwide COVID-19 government interventions. Nat Hum Behav. 2020;4:1303–12. doi:10.1038/s41562-020-01009-0. 33199859

[5] Tabatabaeizadeh SA. Airborne transmission of COVID-19 and the role of face mask to prevent it: a systematic review and meta-analysis. Eur J Med Res. 2021;26:1. doi:10.1186/s40001-020-00475-6. 33388089 PMC7776300

[6] Girum T, Lentiro K, Geremew M, Migora B, Shewamare S. Global strategies and effectiveness for COVID-19 prevention through contact tracing, screening, quarantine, and isolation: a systematic review. Trop Med Health. 2020;48:91. doi:10.1186/s41182-020-00285-w. 33292755 PMC7680824

[7] Islam N, Sharp SJ, Chowell G, Shabnam S, Kawachi I, Lacey B, et al. Physical distancing interventions and incidence of coronavirus disease 2019: natural experiment in 149 countries. BMJ. 2020;370:m2743. doi:10.1136/bmj.m2743. 32669358 PMC7360923

[8] Pinilla J, Barber P, Vallejo-Torres L, Rodriguez-Mireles S, Lopez-Valcarcel BG, Serra-Majem L. The economic impact of the SARS-COV-2 (COVID-19) pandemic in Spain. Int J Environ Res Public Health. 2021;18:4708. doi:10.3390/ijerph18094708. 33925185 PMC8124348

[9] Weaver RG, Hunt ET, Armstrong B, Beets MW, Brazendale K, Turner-McGrievy G, et al. COVID-19 leads to accelerated increases in children’s BMI *z*-score gain: an interrupted time-series study. Am J Prev Med. 2021;61:e161–9. doi:10.1016/j.amepre.2021.04.007. 34148734 PMC8443301

[10] Sorenson SB, Sinko L, Berk RA. The endemic amid the pandemic: seeking help for violence against women in the initial phases of COVID-19. J Interpers Violence. 2021;36:4899–915. doi:10.1177/0886260521997946. 33691528 PMC8064536

[11] Michalska da Rocha B, Rhodes S, Vasilopoulou E, Hutton P. Loneliness in psychosis: a meta-analytical review. Schizophr Bull. 2018;44:114–25. doi:10.1093/schbul/sbx036. 28369646 PMC5768045

[12] Selten JP, Booij J, Buwalda B, Meyer-Lindenberg A. Biological mechanisms whereby social exclusion may contribute to the etiology of psychosis: a narrative review. Schizophr Bull. 2017;43:287–92. doi:10.1093/schbul/sbw180. 28053019 PMC5782499

[13] Wang Q, Xu R, Volkow ND. Increased risk of COVID-19 infection and mortality in people with mental disorders: analysis from electronic health records in the United States. World Psychiatry. 2021;20:124–30. doi:10.1002/wps.20806. 33026219 PMC7675495

[14] Tzur Bitan D, Krieger I, Kridin K, Komantscher D, Scheinman Y, Weinstein O, et al. COVID-19 prevalence and mortality among schizophrenia patients: a large-scale retrospective cohort study. Schizophr Bull. 2021;47:1211–7. doi:10.1093/schbul/sbab012. 33604657 PMC7928567

[15] Harris AF. Influenza as a factor in precipitating latent psychoses and initiating psychoses, with a brief history of the disease and analysis of cases. Boston Med Surg J. 1919;180:610–2. doi:10.1056/nejm191905291802204.

[16] Menninger KA. Influenza psychoses in successive epidemics. J Arch Neurol Psychiatry. 1920;3:57–60.

[17] Parra A, Juanes A, Losada CP, Alvarez-Sesmero S, Santana VD, Marti I, et al. Psychotic symptoms in COVID-19 patients. A retrospective descriptive study. Psychiatry Res. 2020;291:113254. doi:10.1016/j.psychres.2020.113254. 32603930 PMC7311337

[18] Oh TK, Park HY, Song IA. Risk of psychological sequelae among coronavirus disease‐2019 survivors: a nationwide cohort study in South Korea. Depress Anxiety. 2020;38:247–54. doi:10.1002/da.23124.

[19] Keshavan MS. Development, disease and degeneration in schizophrenia: a unitary pathophysiological model. J Psychiatr Res. 1999;33:513–21. doi:10.1016/s0022-3956(99)00033-3. 10628528

[20] McGrath JJ, Feron FP, Burne TH, Mackay-Sim A, Eyles DW. The neurodevelopmental hypothesis of schizophrenia: a review of recent developments. Ann Med. 2003;35:86–93. doi:10.1080/07853890310010005. 12795338

[21] Dalman C, Allebeck P, Gunnell D, Harrison G, Kristensson K, Lewis G, et al. Infections in the CNS during childhood and the risk of subsequent psychotic illness: a cohort study of more than one million Swedish subjects. Am J Psychiatry. 2008;165:59–65. doi:10.1176/appi.ajp.2007.07050740. 18056223

[22] Sutterland AL, Fond G, Kuin A, Koeter MW, Lutter R, van Gool T, et al. Beyond the association. Toxoplasma gondii in schizophrenia, bipolar disorder, and addiction: systematic review and meta-analysis. Acta Psychiatr Scand. 2015;132:161–79. doi:10.1111/acps.12423. 25877655

[23] Simor P, Polner B, Bathori N, Sifuentes-Ortega R, Van Roy A, Albajara Saenz A, et al. Home confinement during the COVID-19: day-to-day associations of sleep quality with rumination, psychotic-like experiences, and somatic symptoms. Sleep. 2021;44:zsab029. doi:10.1093/sleep/zsab029. 33567067 PMC7928634

[24] Mansfield KE, Mathur R, Tazare J, Henderson AD, Mulick AR, Carreira H, et al. Indirect acute effects of the COVID-19 pandemic on physical and mental health in the UK: a population-based study. Lancet Digit Health. 2021;3:e217–30. doi:10.1016/S2589-7500(21)00017-0. 33612430 PMC7985613

[25] Zielasek J, Vrinssen J, Gouzoulis-Mayfrank E. Utilization of inpatient mental health care in the Rhineland during the COVID-19 pandemic. Front Public Health. 2021;9:593307. doi:10.3389/fpubh.2021.593307. 33996706 PMC8119755

[26] Hu W, Su L, Qiao J, Zhu J, Zhou YJPd. COVID-19 outbreak increased risk of schizophrenia in aged adults. Psy ChinaXiv. 2020;10, psych.chinaxiv.org/user/download.htm?id=30325&filetype=pdf [accessed 14 August 2021].

[27] Davis J, Eyre H, Jacka FN, Dodd S, Dean O, McEwen S, et al. A review of vulnerability and risks for schizophrenia: beyond the two hit hypothesis. Neurosci Biobehav Rev. 2016;65:185–94. doi:10.1016/j.neubiorev.2016.03.017. 27073049 PMC4876729

[28] Maynard TM, Sikich L, Lieberman JA, LaMantia AS. Neural development, cell-cell signaling, and the “two-hit” hypothesis of schizophrenia. Schizophr Bull. 2001;27:457–76. doi:10.1093/oxfordjournals.schbul.a006887. 11596847

[29] Chinitz D, Shalev C, Galai N, Israeli A. The second phase of priority setting. Israel’s basic basket of health services: the importance of being explicitly implicit. BMJ. 1998;317:1005–7. 9841023

[30] Hale T, Angrist N, Goldszmidt R, Kira B, Petherick A, Phillips T, et al. A global panel database of pandemic policies (Oxford COVID-19 government response tracker). Nat Hum Behav. 2021;5:529–38. doi:10.1038/s41562-021-01079-8. 33686204

[31] Kodesh A, Goldberg Y, Rotstein A, Weinstein G, Reichenberg A, Sandin S, et al. Risk of dementia and death in very-late-onset schizophrenia-like psychosis: a national cohort study. Schizophr Res. 2020;223:220–6. doi:10.1016/j.schres.2020.07.020. 32807646

[32] Bhaskaran K, Gasparrini A, Hajat S, Smeeth L, Armstrong B. Time series regression studies in environmental epidemiology. Int J Epidemiol. 2013;42:1187–95. doi:10.1093/ije/dyt092. 23760528 PMC3780998

[33] Bernal JL, Cummins S, Gasparrini A. Corrigendum to: interrupted time series regression for the evaluation of public health interventions: a tutorial. Int J Epidemiol. 2020;49:1414. doi:10.1093/ije/dyaa118. 32879971 PMC7750921

[34] Bernal JL, Cummins S, Gasparrini A. Interrupted time series regression for the evaluation of public health interventions: a tutorial. Int J Epidemiol. 2017;46:348–55. doi:10.1093/ije/dyw098. 27283160 PMC5407170

[35] Shadish WR, Cook TD, Campbell DT. Experimental and quasi-experimental designs for generalized causal inference. Boston: Houghton Mifflin; 2002.

[36] Aleman A, Kahn RS, Selten JP. Sex differences in the risk of schizophrenia: evidence from meta-analysis. Arch Gen Psychiatry. 2003;60:565–71.doi:10.1001/archpsyc.60.6.565. 12796219

[37] Werner S, Malaspina D, Rabinowitz J. Socioeconomic status at birth is associated with risk of schizophrenia: population-based multilevel study. Schizophr Bull. 2007;33:1373–8. doi:10.1093/schbul/sbm032. 17443013 PMC2779876

[38] Lipsitch M, Tchetgen E, Cohen T. Negative controls: a tool for detecting confounding and bias in observational studies. Epidemiology. 2010;21:383–8. doi:10.1097/EDE.0b013e3181d61eeb. 20335814 PMC3053408

[39] Hogg RV, Tanis EA. Probability and statistical inference. 9th ed. Upper Saddle River, NJ: Pearson; 2013.

[40] R Core Team. R: a language and environment for statistical computing. Vienna, Austria: R Foundation for Statistical Computing; 2020, https://www.R-project.org/. [accessed 31 December 2020].

[41] Hyndman RJ, Khandakar Y. Automatic time series forecasting: the forecast package for R. J Stat Soft. 2008;27:1.

[42] Wickham H. ggplot2: elegant graphics for data analysis. New York: Springer-Verlag; 2009.

[43] OECD. Israel economic snapshot. Organisation for Economic Co-operation and Development; 2021.

[44] Greener I. Comparing country risk and response to COVID-19 in the first 6 months across 25 organisation for economic co-operation and development countries using qualitative comparative analysis. J Int Comp Soc Policy. 2021;37:1–15. doi:10.1017/ics.2021.6.

