# Supplementary material for: Biopsychosocial exposure to the COVID-19 pandemic and the relative risk of schizophrenia: Interrupted time-series analysis of a nationally representative sample
Source: Eur Psychiatry. 2022 Jan 24;65(1):e7. doi: 10.1192/j.eurpsy.2021.2245 (PMC8853851; doi:10.1192/j.eurpsy.2021.2245)
Supplement: Supplementary file 1 [file S0924933821022458sup001.doc]

**Supplementary material to Biopsychosocial Exposure to the Covid-19 Pandemic and the Relative Risk of Schizophrenia: Interrupted Time-Series Analysis of a Nationally Representative Sample**

Travis-Lumer, Y., Kodesh, A., Goldberg, Y., Reichenberg, A., Frangou, S., Levine, S.Z.,

[Text S1 Socioeconomic status 2](#__RefHeading___Toc94087578)

[Table S1 Forecasted Values and 95% Prediction Intervals, for all Three Scenarios 3](#__RefHeading___Toc94087579)

[Table S2 Relative Risks (RR), the Associated 95% Confidence Intervals (CI), P Values, and Total SSD Rates per 100,000 4](#__RefHeading___Toc94087580)

[Figure S1 A Basic ITS Design 5](#__RefHeading___Toc94087581)

[Figure S2 Residual Autocorrelation - Primary Poisson Model 6](#__RefHeading___Toc94087582)

[Figure S3 Scatterplot and Regression Fitted Values for Males then Females 7](#__RefHeading___Toc94087583)

[Figure S4 Scatterplot and Regression Fitted Values for Different SES Groups 8](#__RefHeading___Toc94087584)

[Figure S5 Scatterplot and Regression Fitted Values for STL and MA Seasonal Decompositions 9](#__RefHeading___Toc94087585)

[Figure S6 Scatterplot and Regression Fitted Values for 15 Day Intervals and Covid-19 10](#__RefHeading___Toc94087586)

[Figure S7 Scatterplot and Regression Fitted Values for Covid-19 and 2014 Gaza War 11](#__RefHeading___Toc94087587)

[Figure S8 Scatterplot and Regression Fitted Values for 15 Day Intervals and Covid-19 + Lockdown 12](#__RefHeading___Toc94087588)

[Figure S9 Scatterplot and Regression Fitted Values restricted to non-Covid-19 Cases 13](#__RefHeading___Toc94087589)

[Supplement References 14](#__RefHeading___Toc94087590)

# Text S1 Socioeconomic status

The demographic covariates were birth date, sex, and city of domicile. Using the city of domicile, we used a neighborhood SES measure developed and validated by the Israeli Central Bureau of Statistics [1]. This SES measure was used in many studies, including schizophrenia research [2-4]. The nation is classified into 1,559 “geographical units,” each area with approximately 2,000 residents. Each area is as homogeneous as possible regarding ethnic background, culture, electronic appliances, and income. Villages with less than 2,000 residents are unclassified to protect anonymity, although such small areas are found to be protective against adverse mental health outcomes, perhaps owing to social cohesion. Each area is given a ranking from 1 to 20 based on information on a wide range of factors, including demographic information, education, the standard of living, employment, and social benefits.[1]

# Table S1 Forecasted Values and 95% Prediction Intervals, for all Three Scenarios

| Time | Scenario 1 | Scenario 2 | Scenario 3 |
| --- | --- | --- | --- |
| March 2021 | 8.09 (7.72, 8.46) | 7.46 (7.13, 7.81) | 6.60 (6.30, 6.91) |
| April 2021 | 7.24 (6.78, 7.73) | 6.81 (6.38, 7.27) | 5.92 (5.55, 6.32) |
| May 2021 | 7.70 (7.11, 8.34) | 7.38 (6.82, 8.00) | 6.28 (5.80, 6.81) |
| June 2021 | 7.52 (6.85, 8.25) | 7.35 (6.70, 8.07) | 6.15 (5.60, 6.75) |
| July 2021 | 7.74 (6.98, 8.59) | 7.72 (6.96, 8.57) | 6.32 (5.69, 7.02) |
| August 2021 | 7.33 (6.53, 8.22) | 7.45 (6.64, 8.35) | 5.99 (5.34, 6.73) |
| September 2021 | 8.23 (7.27, 9.32) | 8.53 (7.53, 9.66) | 6.72 (5.93, 7.62) |
| October 2021 | 6.92 (6.05, 7.91) | 7.30 (6.39, 8.35) | 5.66 (4.95, 6.47) |
| November 2021 | 8.75 (7.59, 10.09) | 9.42 (8.17, 10.86) | 7.14 (6.19, 8.25) |
| December 2021 | 6.74 (5.80, 7.84) | 7.40 (6.36, 8.60) | 5.51 (4.74, 6.42) |

Note. Three forecasts from 1 March 2020 2021 to 1 December 2021. Abbreviations Scenario (1) no ongoing effects of the Covid-19 pandemic; (2) ongoing effects of the Covid-19 pandemic; and (3) based on the intervals before and during the Covid-19 pandemic. As a reference, forecasts for December 2021 may be contrasted to past observed values for December 2019 (6.75) and December 2020 (5.31). Multi-step prediction intervals (PIs) were calculated using the multi-step forecast standard deviation for the drift method[5].

# Table S2 Relative Risks (RR), the Associated 95% Confidence Intervals (CI), P Values, and Total SSD Rates per 100,000

| Model | RR (95% CI) | P value | SSD rate (95% CI) |
| --- | --- | --- | --- |
| Primary Poisson model | 0.81 (0.73, 0.91) | P<0.001 | 585.31 (567.97, 603.06) |
| Restricted to males | 0.86 (0.76, 0.99) | 0.03 | 770.94 (742.96, 799.71) |
| Restricted to females | 0.71 (0.59, 0.87) | P<0.001 | 396.45 (376.28, 417.41) |
| Restricted to low SES | 0.80 (0.66, 0.95) | 0.01 | 701.43 (665.66, 738.64) |
| Restricted to medium SES | 0.86 (0.74, 1.00) | 0.05 | 571.17 (548.03, 595.03) |
| Restricted to high SES | 0.62 (0.39, 1.00) | 0.05 | 310.41 (276.05, 347.86) |
| Loess STL | 0.82 (0.73, 0.91) | P<0.001 | 585.31 (567.97, 603.06) |
| Moving average | 0.82 (0.73, 0.91) | P<0.001 | 585.31 (567.97, 603.06) |
| 15-day aggregation | 0.82 (0.73, 0.91) | P<0.001 | 585.31 (567.97, 603.06) |
| Covid-19 + Gaza war | 0.81 (0.73, 0.91) | P<0.001 | 585.31 (567.97, 603.06) |
| Covid-19 + lockdown | 0.96 (0.79, 1.15) | 0.62 | 585.31 (567.97, 603.06) |
| No Covid-19 cases | 0.82 (0.74, 0.92) | P<0.001 | 590.24 (572.75, 608.14) |

Note. Abbreviations. SSD, Schizophrenia Spectrum Disorders. Loess STL and moving averages (MA) are both types of seasonal decompositions. All models, except for “Covid-19 + lockdown,” compute the RR based on dividing the predicted values for the Covid-19 exposure interval with their counterfactual values. The “Covid-19 + lockdown” model computes the RR by dividing the predicted values of Covid-19 + lockdown with the predicted values of Covid-19 alone (with no lockdown).

# Figure S1 A Basic ITS Design


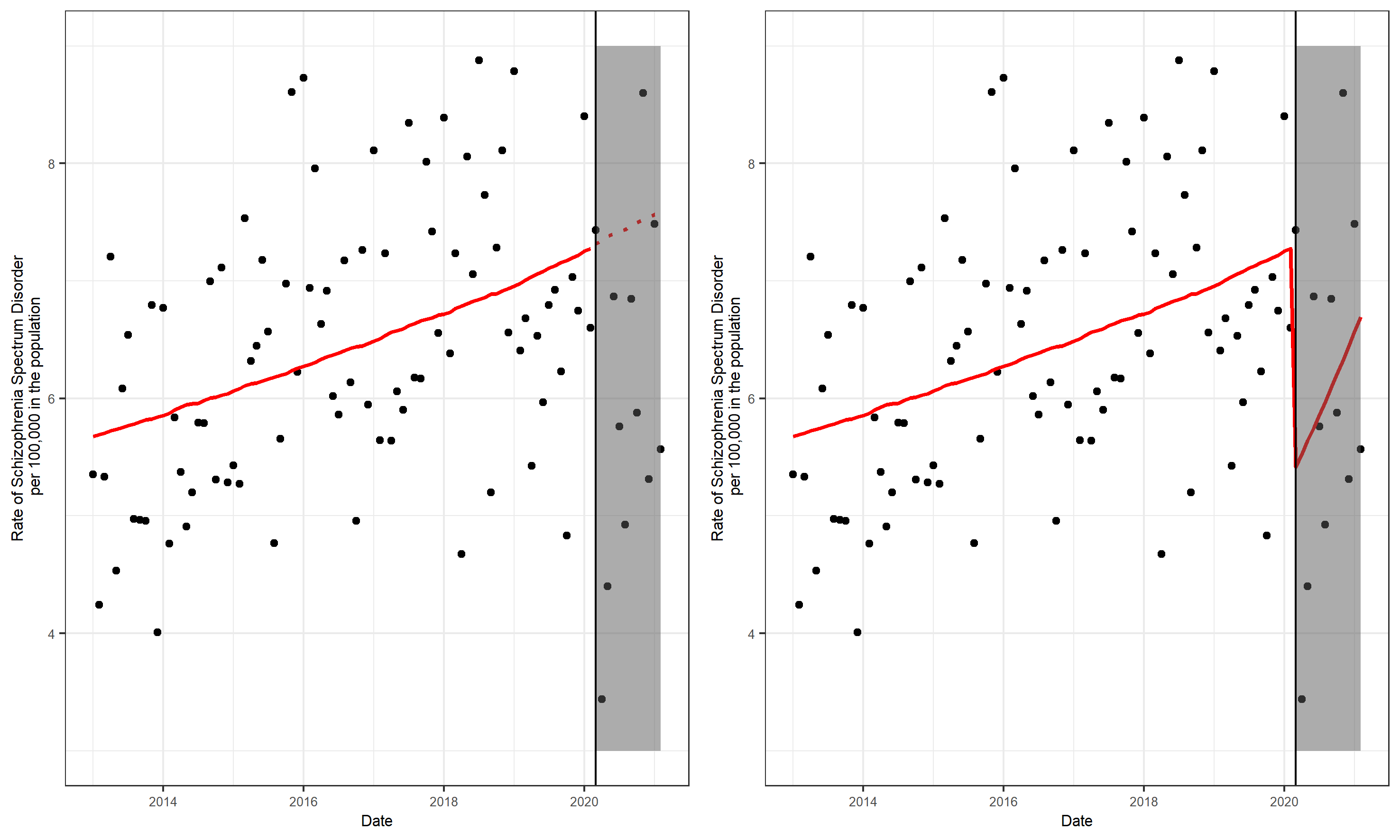


Note. Scatter plot of the monthly rate of SSD. White background: pre-Covid-19 period, grey background: post-Covid-19 period. Left: The continuous red line is the fitted pre-Covid-19 trend, and the dotted red line is the predicted trend had Covid-19 not have occurred (counterfactual). Right: The continuous red line is the fitted pre and post Covid-19 trend.

# Figure S2 Residual Autocorrelation - Primary Poisson Model


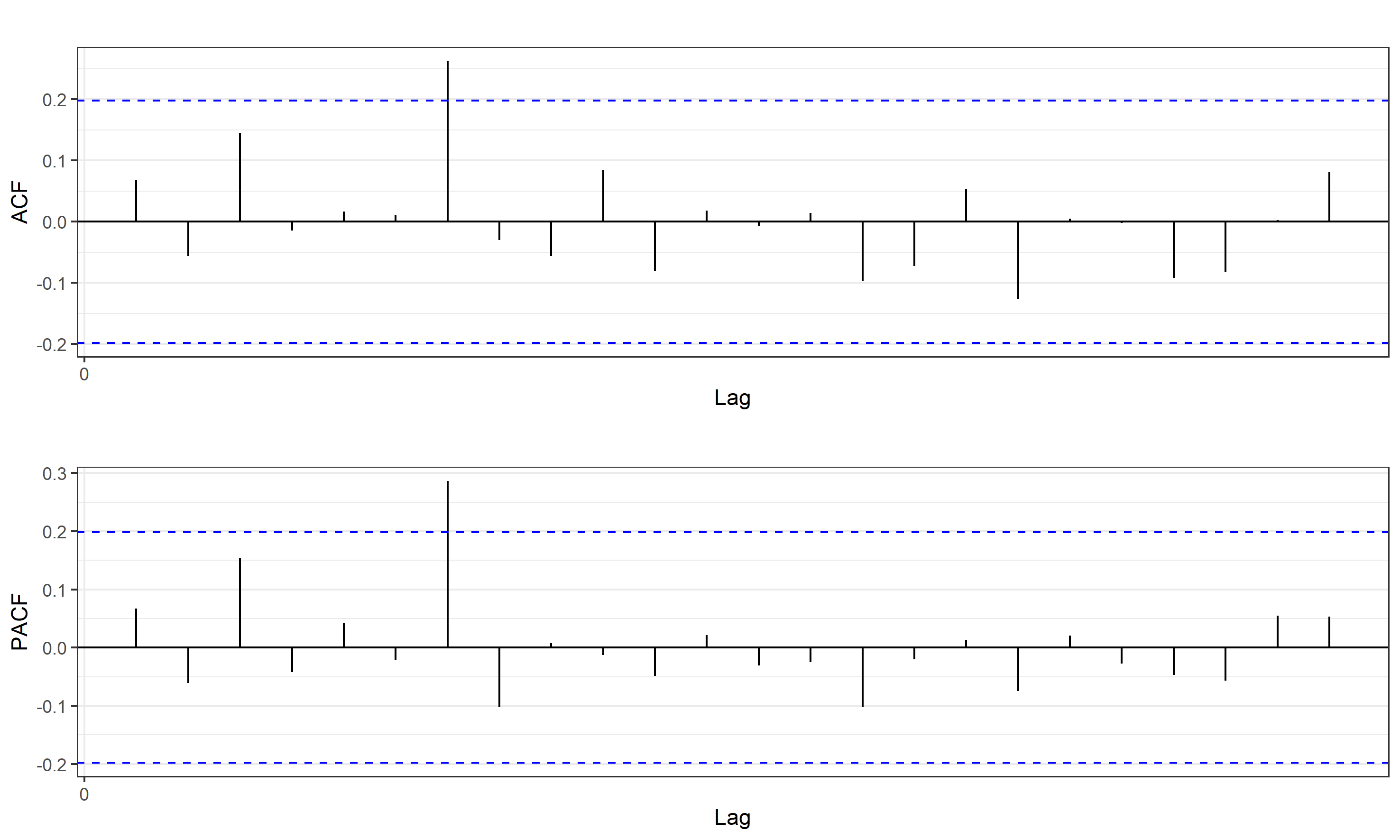


Note. Abbreviations. ACF, autocorrelation function, PACF, partial autocorrelation function. Residual autocorrelation top, residual partial autocorrelation lower panel. The blue dashed lines represent a 95% confidence interval. Any correlation within the blue dashed lines is not considered different than zero. In this case, we observe that all but one of the ACFs and PACFs are within the confidence interval and hence do not violate the white noise assumption stating the residuals are uncorrelated.

# Figure S3 Scatterplot and Regression Fitted Values for Males then Females


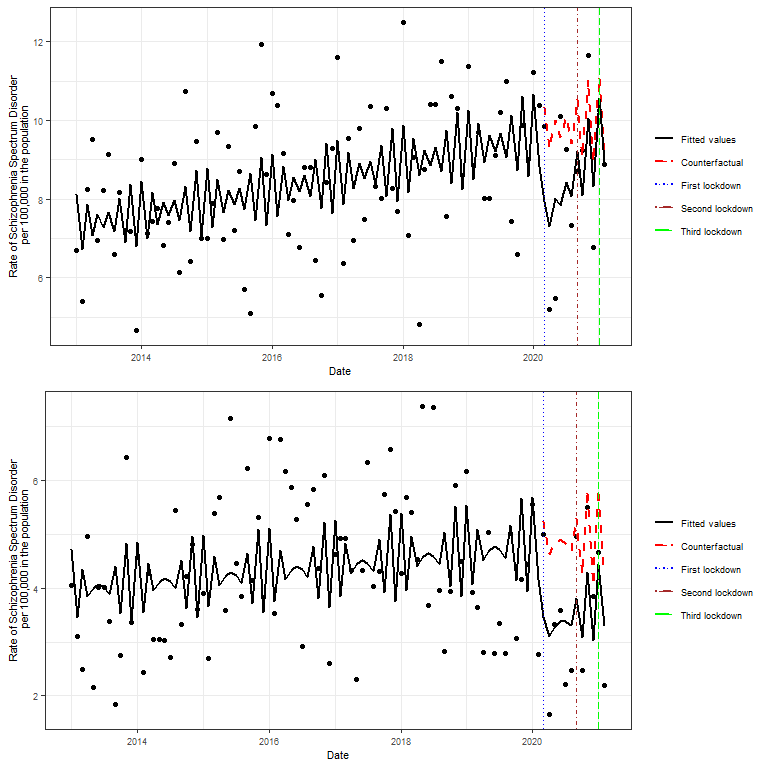


Note. Males top panel, females lower panel. Scatter plot of the monthly rate of SSD, together with the regression fitted values (in black), and the counterfactual (in red). The blue horizontal line marks the first lockdown in Israel, the brown horizontal line marks the month of the second lockdown in Israel, and the green horizontal line marks the month of the third lockdown in Israel.

# Figure S4 Scatterplot and Regression Fitted Values for Different SES Groups


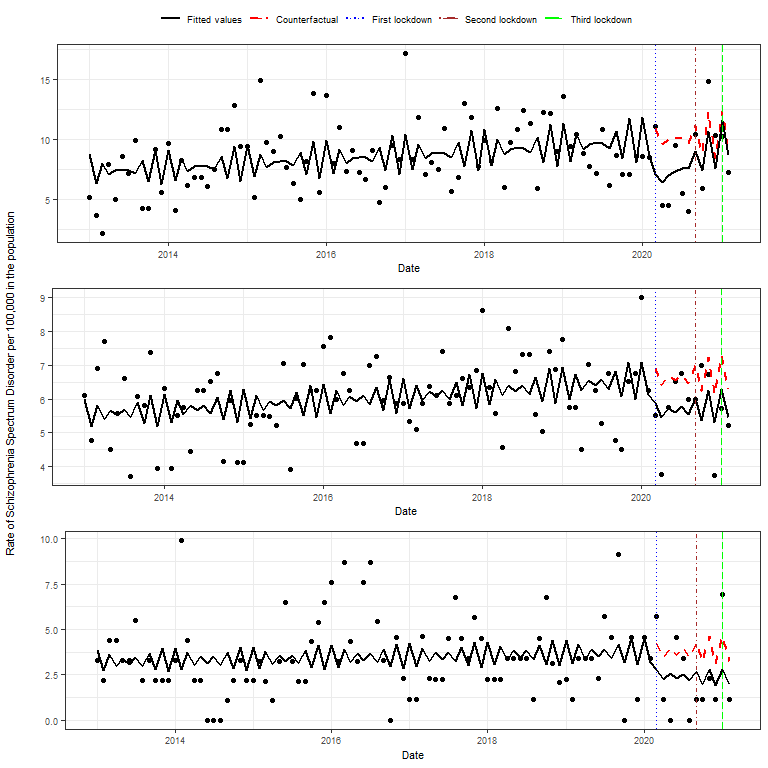


Note. Top low SES, middle medium SES, bottom high SES. Scatter plot of the monthly rate of SSD, together with the regression fitted values (in black), and the counterfactual (in red). The blue horizontal line marks the first lockdown in Israel, the brown horizontal line marks the month of the second lockdown in Israel, and the green horizontal line marks the month of the third lockdown in Israel.

# Figure S5 Scatterplot and Regression Fitted Values for STL and MA Seasonal Decompositions


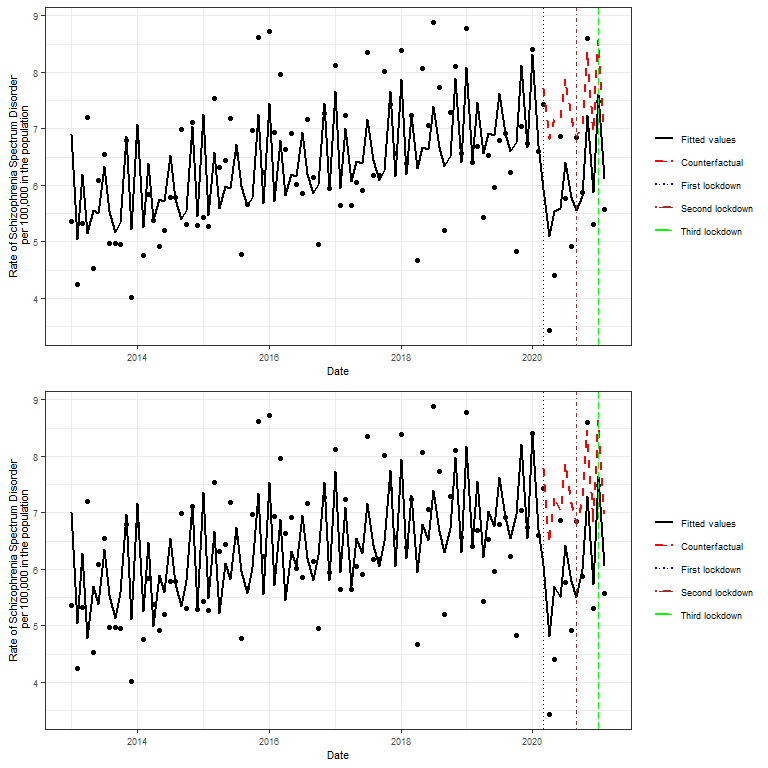


Note. STL seasonal decomposition top, MA seasonal decomposition lower panel. Scatter plot of the monthly rate of SSD, together with the regression fitted values (in black), and the counterfactual (in red). The blue horizontal line marks the first lockdown in Israel, the brown horizontal line marks the month of the second lockdown in Israel, and the green horizontal line marks the month of the third lockdown in Israel.

# Figure S6 Scatterplot and Regression Fitted Values for 15 Day Intervals and Covid-19


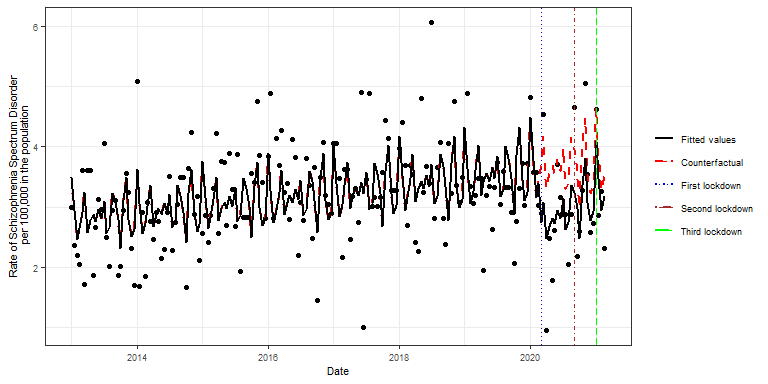


Note. Scatter plot of biweekly rate of SSD, together with the regression fitted values (in black), and the counterfactual (in red). The blue horizontal line marks the first lockdown in Israel, the brown horizontal line marks the month of the second lockdown in Israel, and the green horizontal line marks the month of the third lockdown in Israel.

# Figure S7 Scatterplot and Regression Fitted Values for Covid-19 and 2014 Gaza War


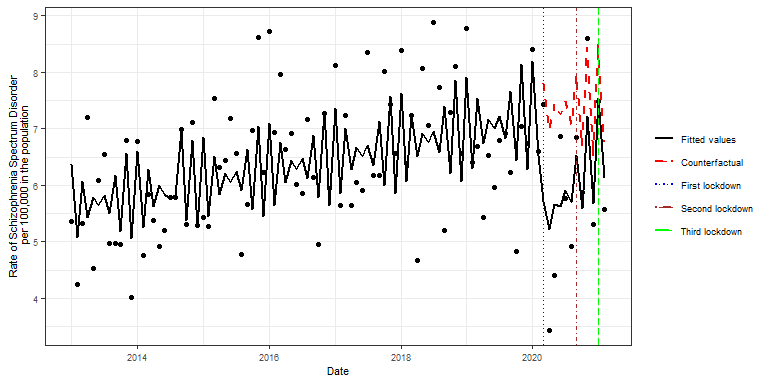


Note. Scatter plot of the monthly rate of SSD, together with the regression fitted values (in black) and the counterfactual (in red). The blue horizontal line marks the first lockdown in Israel, the brown horizontal line marks the month of the second lockdown in Israel, and the green horizontal line marks the month of the third lockdown in Israel.

# Figure S8 Scatterplot and Regression Fitted Values for 15 Day Intervals and Covid-19 + Lockdown


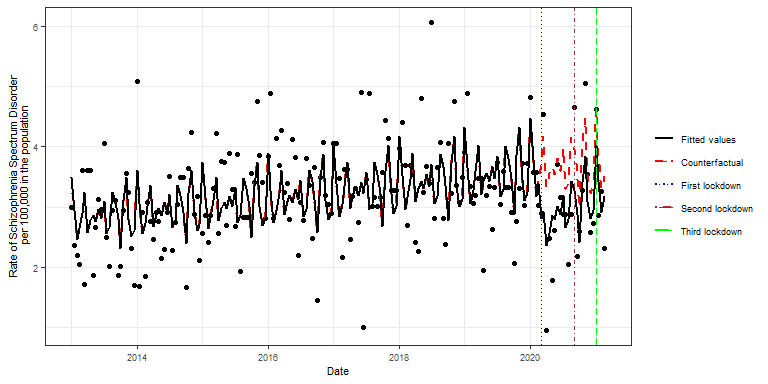


Note. Scatter plot of the biweekly rate of SSD, together with the regression fitted values (in black), and the counterfactual (in red). The blue horizontal line marks the first lockdown in Israel, the brown horizontal line marks the month of the second lockdown in Israel, and the green horizontal line marks the month of the third lockdown in Israel.

# Figure S9 Scatterplot and Regression Fitted Values restricted to non-Covid-19 Cases


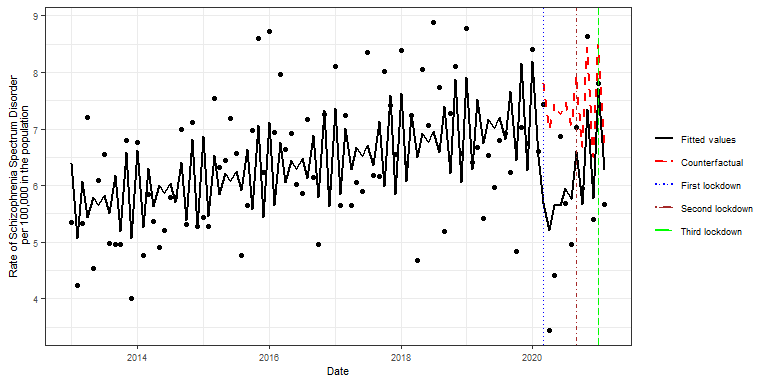


Note. Scatter plot of the monthly rate of SSD, together with the regression fitted values (in black), and the counterfactual (in red). The blue horizontal line marks the first lockdown in Israel, the brown horizontal line marks the month of the second lockdown in Israel, and the green horizontal line marks the month of the third lockdown in Israel.

# Supplement References

[1] Central Bureau of Statistics. Demographic Characteristics of the Population in Localities and Statistical Areas. Jerusalem, Israel 1995

[2] Levine SZ, Levav I, Goldberg Y, Pugachova I, Becher Y, Yoffe R. Exposure to genocide and the risk of schizophrenia: a population-based study. Psychol Med. 2016;46:855-63. 10.1017/S0033291715002354

[3] Levine SZ, Levav I, Yoffe R, Becher Y, Pugachova I. Genocide Exposure and Subsequent Suicide Risk: A Population-Based Study. PLoS One. 2016;11:e0149524. 10.1371/journal.pone.0149524

[4] Goldberg S, Fruchter E, Davidson M, Reichenberg A, Yoffe R, Weiser M. The relationship between risk of hospitalization for schizophrenia, SES, and cognitive functioning. Schizophr Bull. 2011;37:664-70. 10.1093/schbul/sbr047

[5] Hyndman RJ, Khandakar Y. Automatic Time Series Forecasting: The forecast Package for R. Journal of Statistical Software. 2008;1.
